# Robustaflavone Isolated from *Nandina domestica* Using Bioactivity-Guided Fractionation Downregulates Inflammatory Mediators

**DOI:** 10.3390/molecules24091789

**Published:** 2019-05-08

**Authors:** Ara Jo, Hyun Ji Yoo, Mina Lee

**Affiliations:** College of Pharmacy, Sunchon National University, 255 Jungangno, Suncheon-si 57922, Jeonnam, Korea; coo123mm@naver.com (A.J.); youhj915@naver.com (H.J.Y.)

**Keywords:** *Nandina domestica*, bioactivity-guided isolation, robustaflavone, inflammatory mediators, inflammatory bowel disease

## Abstract

*Nandina domestic*a (Berberidaceae) has been used in traditional medicine for the treatment of cough. This plant is distributed in Korea, Japan, China, and India This study aimed to investigate the anti-inflammatory phytochemicals obtained from the *N. domestica* fruits. We isolated a biflavonoid-type phytochemical, robustaflavone (R), from *N. domestica* fruits through bioactivity-guided fractionation based on its capacity to inhibit inflammation. The anti-inflammatory mechanism of R isolated from *N. domestica* has not yet been studied. In the present study, we evaluated the anti-inflammatory activities of R using lipopolysaccharide (LPS)-stimulated RAW 264.7 macrophages. We have shown that R reduces the production of nitric oxide (NO), pro-inflammatory cytokine interleukin-1 beta (IL-1β), and IL-6. Western blot analysis showed that R suppresses the expression of inducible nitric oxide synthase (iNOS) and cyclooxygenase-2 (COX-2), and downregulates the expression of LPS-induced nuclear factor-kappa B (NF-κB) and the phosphorylation of extracellular-regulated kinases (pERK 1/2). Moreover, R inhibited IL-8 release in LPS-induced human colonic epithelial cells (HT-29). These results suggest that R could be a potential therapeutic candidate for inflammatory bowel disease (IBD).

## 1. Introduction

The inflammatory response is a defense strategy against tissue injuries and infections, and it is essential for maintaining homeostasis in normal tissues [1]. Inflammatory diseases are associated with the overexpression of nitric oxide (NO) and cytokines, such as interleukin-1 beta (IL-1β) and IL-6 [2], and stimulation of the macrophages that produce them during the inflammatory response [3]. During inflammation, cyclooxygenase-2 (COX-2) catalyzes the conversion of arachidonic acid to prostaglandin H2 (PGH2), a precursor of various inflammatory mediators such as prostaglandin E2 (PGE2) [4]. Many human diseases arise from the overexpression of these inflammatory mediators, and, thus, reducing the expression of these mediators might be an effective way to treat inflammatory diseases [5]. 

Nuclear factor-kappa B (NF-κB) plays a key role in the inflammatory response as it can induce the transcription of pro-inflammatory genes [6]. In addition, it is known that NF-κB plays an important role in the production of inflammatory mediators such as COX-2 and inducible nitric oxide synthase (iNOS) [7]. NF-κB is a protein complex made up of p50 and p65 subunits and it is regulated by Iκb [8]. It was reported that activation of NF-κB can induce mitogen-activated protein kinase (MAPK) and extracellular-regulated kinase (ERK) signaling cascades [9], as well as LPS-stimulated activation of macrophages, which, in turn, induces ERK1/2 [10]. MAPKs, such as ERK1/2, are overexpressed in inflammatory diseases [11]. Inhibitors of ERK1/2 have been shown to reduce the secretion of eosinophilic pro-inflammatory cytokines, as well as degranulation and differentiation in vitro [12]. These factors are considered as key targets for inhibiting inflammatory diseases [13]. 

Inflammatory bowel disease (IBD) is a chronic recurrent disease that is caused by epithelial damage and endocrine inflammation of the intestine [14]. It has been seen that IBD and chronic inflammation of the large bowel are related to the development of colon cancer [15]. IL-8 is an important cytokine that activates and recruits neutrophils. Patients with Crohn’s disease (CD) and ulcerative colitis (UC) have IL-8-expressing cells in the intestinal mucosal region [16]. It has also been shown that IL-8 is associated with IBD. Additionally, pro-inflammatory cytokines such as IL-6 are involved in the pathogenesis of chronic mucosal inflammation during IBD and hyperactivation of this inflammatory response results in colon tissue damage [17]. Therefore, it is crucial to find a factor that can downregulate IL-8 and IL-6 and, hence, be used as a therapeutic candidate for IBD patients.

*Nandina domestica* is a species of flowering plant that belongs to the family Berberidaceae. Some of the known chemicals isolated from *N. domestica* are *O*-methylapallidine, sinoacutine, jatrorrhizine, palmatine, berberine, (*S*)-camegine, (*R*)-camegine, higenamine, 24-methylenecycloartanone 13-carboxylic acid, (*E,E*)-terrestribisamind, (*Z,Z*)*-*terrestribisamind, *4*-β-d-glucopyranosyloxy benzoic acid, and (*E*)-4-(3,4,5-trihydroxy-6-(hydroxymethyl)tetrahydro-2*H*-pyran-2-yloxy)benzyl 3-(3,4-dihydroxyphenyl)acrylate. *N. domestica* has been found to be effective in the treatment of dermatophytic infections [18] and solitary mastocytoma [19]. In addition, *N. domestica* fruits are known to have antioxidant [20] and anti-inflammatory properties [21]. It has also been known to relax tracheal smooth muscles [22] and, hence, is used to treat a cough [23]. However, the biflavonoid-type phytochemical robustaflavone (R), isolated from *N. domestica*, has not been adequately researched. Therefore, we decided to explore the anti-inflammatory effects of R and the related mechanisms.

## 2. Results 

### 2.1. Bioactivity-Guided Isolation of an Active Phytochemical from N. domestica Fruits

**Figure 1:**
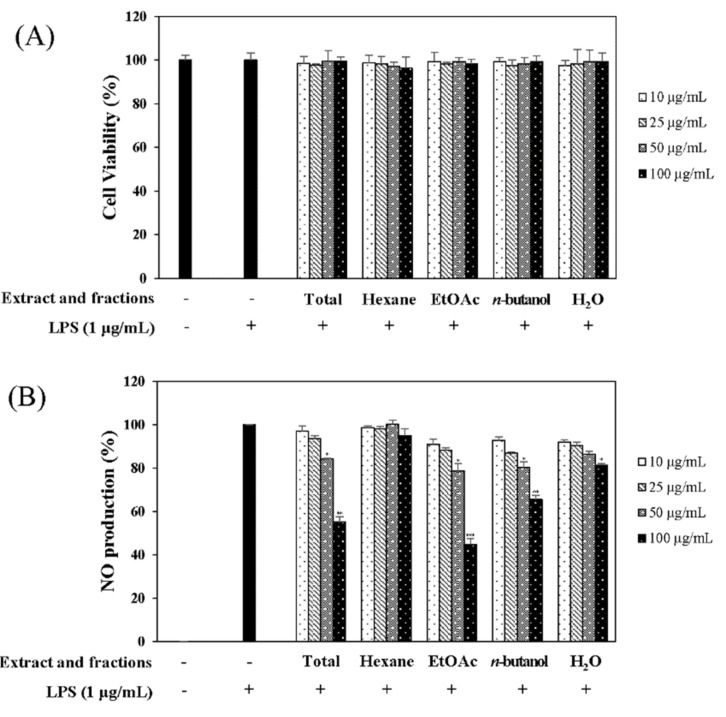
Effects of the total extract and extract fractions of *Nandina domestica* fruits on cell viability (**A**) and nitric oxide production (**B**). RAW 264.7 cells were cultured in the presence of the extracts for 1 h and stimulated with lipopolysaccharide (LPS) (1 µg/mL) for 16 h. Cell viability and NO production were detected using the MTT (3-[4,5-dimethylthiazol-2-yl]-2,5 diphenyl tetrazolium bromide) assay and the Griess reagent, respectively. Nitrite concentrations from the non-treated and LPS-treated controls were 0.81 ± 0.1 μM and 17.2 ± 0.2 μM, respectively. Each determination was made in triplicate. The data are represented as the mean ± SD. * *p* < 0.05, ** *p* < 0.01, *** *p* < 0.001 vs. LPS-treated group.

**Figure 2:**
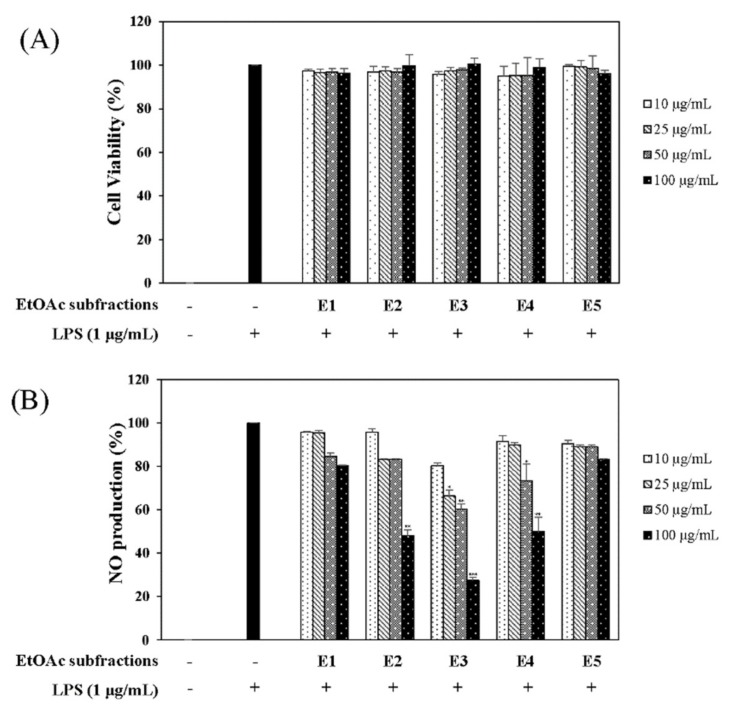
Effects of ethyl acetate (EtOAc) subfractions (E1–E5) on cell viability (**A**) and NO production (**B**). RAW 264.7 cells were cultured in the presence of the *N. domestica* extract for 1 h and stimulated with LPS (1 µg/mL) for 16 h. Cell viabilities and NO production were detected using the MTT assay and the Griess reagent, respectively. Nitrite concentrations of non-treated and LPS-treated controls were 0.8 ± 0.04 μM and 17.5 ± 0.3 μM, respectively. Each experiment was performed in triplicate. The data are represented as the mean ± SD. * *p* < 0.05, ** *p* < 0.01, *** *p* < 0.001 vs. LPS-treated group.

**Figure 3:**
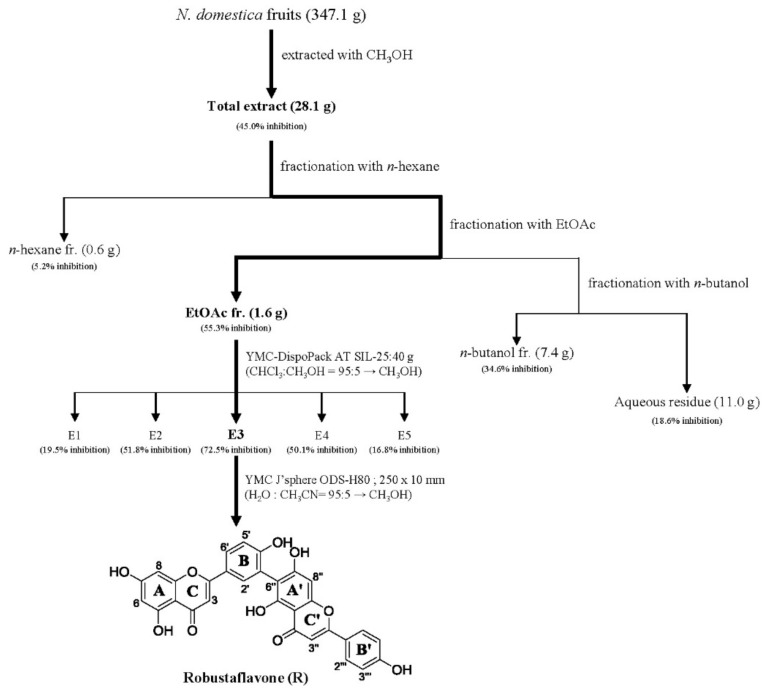
Schematic representation of the isolation of robustaflavone (R) from *N*. *domestica* fruits using bioactivity-guided fractionation. Bioactivity-guided fractionation of *N. domestica* fruit was performed as shown in the schematic representation and resulted in the isolation and identification of R. Fractionation was guided by assessing the inhibitory effect of R on NO production at concentrations of 100 μg/mL without any cytotoxicity. At each level of fractionation, all the fractions generated were tested simultaneously and were compared to the crude extract.

In the quest for natural anti-inflammatory products for the treatment of IBD, we evaluated the inhibitory effect of *N. domestica* fruit extracts and the fractions on inflammation in LPS-stimulated RAW 264.7 cells by measuring the NO production after pretreatment. NO production and cell cytotoxicity were measured by the Griess reagent and MTT (3-[4,5-dimethylthiazol-2-yl]-2,5 diphenyl tetrazolium bromide) assay, respectively. The methanolic extract of the *N. domestica* fruit could significantly inhibit NO production (45% of the LPS group at 100 μg/mL, *p* < 0.001) (Figure 1). Based on bioactivity-guided fractionation, the extract was partitioned into fractions using *n*-hexane, ethyl acetate (EtOAc), *n*-butanol, and H_2_O, depending on the solvent polarity. All the fractions did not show cytotoxicity and had concentration-dependent inhibitory effects on the NO production, except the *n*-hexane fraction (Figure 1). Among them, the EtOAc fraction showed the most potent inhibitory activity (55.3% of the LPS group at 100 μg/mL; *p* < 0.001) and, hence, was subjected to further isolation. The EtOAc fraction was separated using liquid chromatography into five subfractions (E1 to E5). The effects of the EtOAc subfractions (E1 to E5) on NO production and cytotoxicity were evaluated in LPS-induced RAW 264.7 cells. The EtOAc subfractions did not affect cell viability and had an inhibitory effect on NO production in a concentration-dependent manner (Figure 2). The E3 fraction had a greater inhibitory effect than the other fractions, which was about 72.5% at a concentration of 100 μg/mL. Therefore, a single compound was isolated from the most active subfraction, E3, using C_18_ HPLC (high-performance liquid chromatography)chromatography for 72 h. The purity of R was 95% by normalization of the peak areas detected by HPLC–DAD analysis. The ^1^H NMR spectrum of the compound showed chemical shift signals corresponding to aromatic compounds in the region δ H 6–8 ppm. Two doublet signals at δ 6.30 (1H, d, *J* = 2.0 Hz, H = 8) and 6.17 (1H, d, *J* = 1.2 Hz, H-6) could be assigned to the A ring. The signals at δ 7.93 (1H, dd, *J* = 2, 8.4 Hz, H-6′), 8.08 (1H, d, *J* = 1.6 Hz, H-2′), and 7.12 (1H, d, *J* = 7.2 Hz, H-5′) revealed a coupling in the 3′,4′-bisubstituted B ring indicating that C-3′ or C-4′ would be the position of linkage of the two flavonoid units. Two doublet signals at δ 7.60 (2H, d, *J* = 8.8 Hz, H-2′′′, 6′′′) and 6.67 (2H, d, *J* = 8.4 Hz, H-3′′′, 5′′′) could be assigned to the spin system of the symmetric *para*-substituted B′ ring. The singlet signals at δ 6.64 (1H, s, H-3′′) and 6.62 (1H, s, H-3) were identified as the protons H-3′′, H-3 in ring C′ and C. Further, the singlet at δ 6.33 appeared in the A’ ring (1H, s, H-8′′). This also proves the ring A′ contains substituents at position C-6′′, which have interflavonoid linkage with C-3′′ or C-4′′. The ^1^H-^1^H COSY correlations of δ 7.60 (2H, d, *J* = 8.8 Hz, H-2′′′, 6′′′) and 6.67 (2H, d, *J* = 8.4 Hz, H-3′′′, 5′′′) and δ 7.93 (1H, dd, *J* = 2, 8.4 Hz, H-6′) to 7.12 (1H, d, *J* = 7.2 Hz, H-5′) enabled us to construct the structure of the compound as shown in Figure 3. The HMBC analysis showed the correlations of H-2′ and H-8′′ to C-7′′. Additionally, we identified correlations for H-6′′′ to C-2′′ and H-6′ to C-2′, respectively. Based on this data, the compound was confirmed as robustaflavone [24]. Bioactivity-guided fractionation from *N. domestica* by inhibition of NO production at a concentration of 100 μg/mL without any cytotoxicity was performed according to the schematic representation in Figure 3 and resulted in the isolation and identification of R. 

### 2.2. Effects of R on NO Production and Cytotoxicity in LPS-Induced Mouse Macrophage Cells

**Figure 4:**
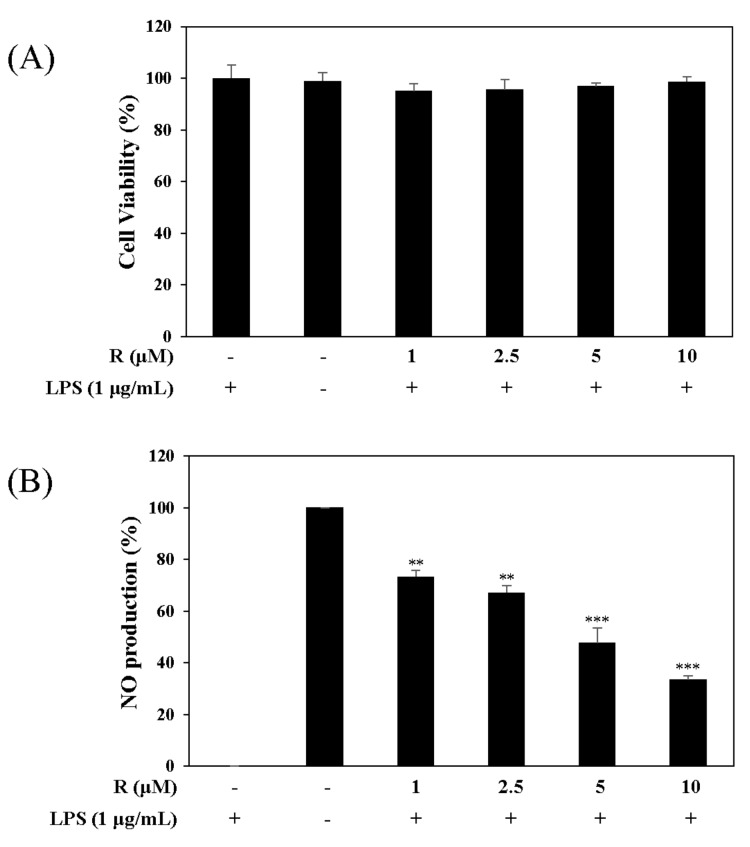
Effects of R on cell viability (**A**) and NO production (**B**). RAW 264.7 cells were treated with R for 1 h and stimulated with LPS (1 µg/mL) for 16 h. Cell viability and NO production were detected using the MTT assay and Griess reagent, respectively. Nitrite concentrations of non-treated and LPS-treated controls were 0.8 ± 0.1 μM and 17.3 ± 0.2 μM, respectively. Each experiment was performed in triplicate. The data are represented as the mean ± SD. * *p* < 0.05, ** *p* < 0.01, *** *p* < 0.001 vs. LPS-treated group.

We tested the inhibitory effect of R on NO production using the Griess reagent and measured cell viability using the MTT assay. The results showed that R did not affect cell viability and reduced LPS-induced NO production in a concentration-dependent manner in RAW 264.7 cells at concentrations ranging from 1 µM to 10 µM (Figure 4).

### 2.3. Effects of R on the Expression of iNOS and COX-2 in LPS-Induced Mouse Macrophage Cells

**Figure 5:**
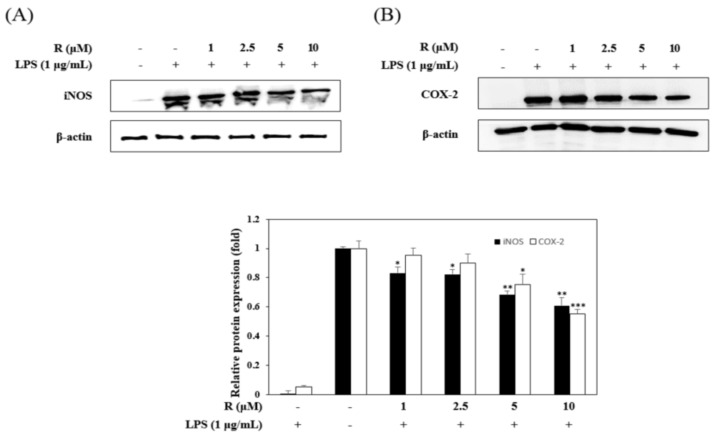
Effects of R on iNOS and cyclooxygenase COX-2 expression. RAW 264.7 cells were incubated in the presence of R for 1 h and then stimulated with LPS (1 µg/mL) for 16 h. The expression of iNOS (**A**), COX-2 (**B**), and β-actin in the LPS-induced cells was determined by Western blot analysis. The relative density was calculated as the ratio of the level of each protein expressed to the level of β-actin. * *p* < 0.05, ** *p* < 0.01, *** *p* < 0.001 vs. LPS-treated group.

To confirm the anti-inflammatory effects, we evaluated the inhibitory effects of R on the expression of iNOS and COX-2 in LPS-stimulated RAW264.7 cells using Western blot analysis. The LPS-induced group dramatically overexpressed iNOS and COX-2, while the R pretreated group had significantly reduced expression of iNOS and COX-2 (Figure 5).

### 2.4. Effects of R on the Expression of Pro-Inflammatory Cytokines, IL-1β and IL-6, in LPS-Induced Mouse Macrophage Cells 

**Figure 6:**
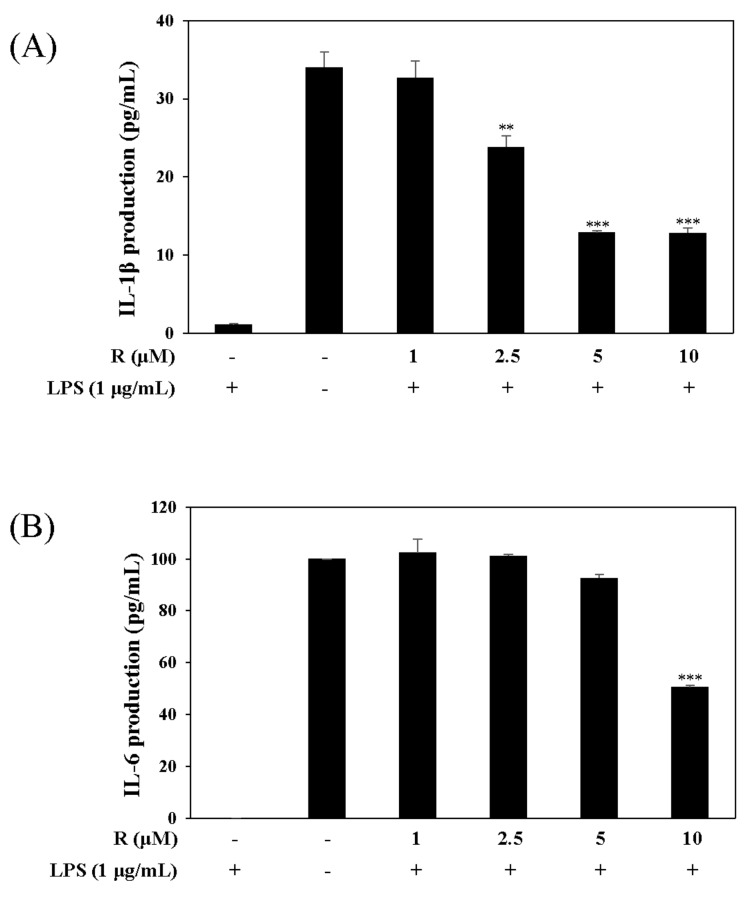
Effect of R on the expression of proinflammatory cytokines, IL-1β and IL-6. RAW 264.7 cells were pretreated with R for 1 h, then incubated with LPS (1 µg/mL) for 16 h. The levels of IL-1β (**A**) and IL-6 (**B**) in culture media were measured by ELISA. The data are represented as the mean ± SD. * *p* < 0.05, ** *p* < 0.01, *** *p* < 0.001 vs. LPS-treated group.

Pro-inflammatory cytokines such as IL-1β and IL-6 are recognized as key markers of inflammation. To determine the potential anti-inflammatory effects of R, we conducted experiments related to transcription factor signaling. These experiments were performed using the harvested supernatant from the culture media of LPS-stimulated RAW 264.7 cells. The group treated with R reduced the expression of pro-inflammatory cytokines, IL-1β and IL-6, unlike the group without R (Figure 6).

### 2.5. Effect of R on the Activation of NF-κB and pERK1/2 in LPS-Induced Mouse Macrophage Cells

**Figure 7:**
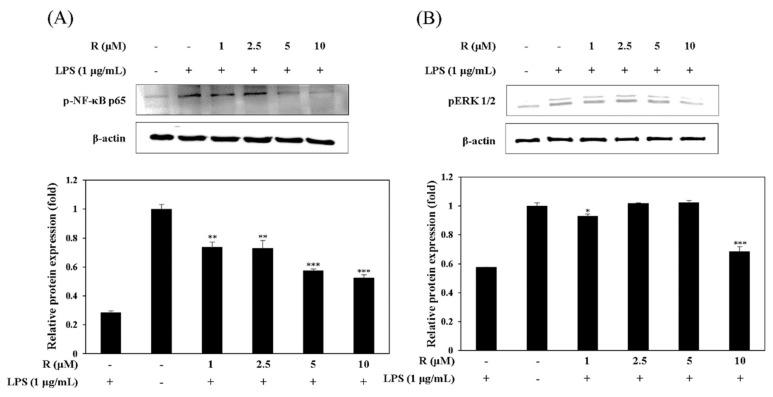
Effect of R on NF-κB activation (**A**) and pERK1/2 expression (**B**). RAW 264.7 cells were cultured in the presence of R for 16 h and stimulated with LPS (1 µg/mL) for 1 h under serum-free conditions. The expression of NF-κB, p65, and pERK1/2 was detected by Western blot analysis. The results presented are representative of three independent experiments. The relative density was calculated as the ratio of the expression level of each protein with that of β-actin. * *p* < 0.05, ** *p* < 0.01, *** *p* < 0.001 vs. LPS-treated group.

It has been known that the expression of NF-κB is related to NO production in LPS-induced mouse macrophage cells [25]. Thus, we studied the effect of R on LPS-stimulated NF-κB activation in mouse macrophage cells using Western blot assay. The results showed that R attenuated the release of NF-κB in the LPS-induced cells (Figure 7A). MAP kinases have an important role in the modulation of cell growth and differentiation as well as the regulation of cytokines and cellular stress responses [26]. We investigated the inhibitory effect of pERK1/2, one of the MAP kinases, in LPS-induced mouse macrophage cells. The data showed that R is involved in the inhibition of pERK1/2 in RAW 264.7 cells (Figure 7B).

### 2.6. Effect of R on IL-8 Production in LPS-Induced HT-29 Colon Epithelial Cells 

**Figure 8:**
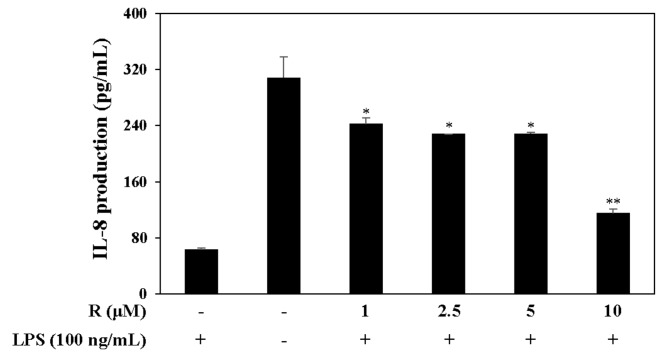
Effect of R on IL-8 expression. HT-29 colon epithelial cells were pretreated with R for 2 h and stimulated with LPS (100 ng/mL) for 12 h. The levels of IL-8 in the culture media were measured by ELISA. The data are represented as the mean ± SD. * *p* < 0.05, ** *p* < 0.01, vs. LPS-treated group.

Pro-inflammatory cytokines such as interleukin-8 (IL-8), which is associated with IBD, is secreted from LPS-stimulated HT-29 cells [27]. Therefore, we measured the levels of the pro-inflammatory cytokine IL-8 by harvesting the supernatant of LPS-induced (100 ng/mL) HT-29 cells. The cells were pretreated with R in serum-free media, and the results confirm that R reduced IL-8 levels in the LPS-induced HT-29 cells (Figure 8).

## 3. Discussion

The prevalence rate of domestic IBD patients is increasing year by year, and IBD patients are increasing rapidly all over the world. IBD is a disorder of various causes that presents as diverse phenotypes, severity, and clinical behaviors [28]. Thereforethis study investigated an anti-inflammatory mechanism on R isolated from a medicinal plant with IBD as a target. 

The bioactivity-guided fractionation approach was efficiently used to screen various plant materials and isolate an active phytochemical as a therapeutic candidate. To search the anti-inflammatory candidates against IBD, the process was guided by the assessment of inhibition on NO production in LPS-induced RAW 264.7 cells under no cytotoxicity. All extracts, fractions, and subfractions generated at each fractionation level were simultaneously tested. Column chromatographic separation targeting the most significant NO-reducing fraction led to the isolation of an active phytochemical. R was newly isolated from *N. domestica*. 

The expression of iNOS increases production of NO, causing the pathogenesis of various inflammatory diseases [29]. Also, COX-2 of inducible isoforms regulates the inflammatory mediator PGE2 [30]. We investigated the cell viability of RAW 264.7 cells to find the concentration of R to be used in all of the experiments. The results exhibited no toxic effect in the concentration range used (1 to 10 µM) (Figure 4A). Also, it was found that at the selected concentrations, R inhibited the production of the inflammatory regulatory mediator NO (Figure 4B). These results show that R downregulates iNOS (Figure 5A). In addition, R downregulated COX-2, relevant to the inflammatory mediator PGE2 (Figure 5B). 

Gene expression, apoptosis, cell growth, and the controlling cellular responses are regulated by MAPKs, which play a key role in the control of signaling pathways [31]. The ERK pathway is a molecular target for the development of drugs. Inhibitors of MAPK, including ERK, have been developed to treat human diseases [32]. Moreover, NF-κB, a transcription factor, regulates various cellular responses. NF-κB regulates the transcription that controls various cellular responses and it controls many pro-inflammatory cytokines. The upregulated expression of these cytokines has been associated with the pathogenesis of IBD [33]. Also, downregulation of NF-κB activation leads to a reduction in iNOS and NO expression [34]. We investigated the molecular mechanism of R in the inflammatory response. The effect of R on the activation of NF-κB and pERK1/2 in LPS-exposed RAW 264.7 cells was assessed. The results show that R reduced NF-κB and pERK1/2 activation (Figure 7A,B).

The pathogenesis of IBD is implicated in pro-inflammatory cytokines such as IL-1β [34,35]. IL-1β and IL-8 are higher in patients with Crohn’s colitis [36]. The IL-8 levels in tissues of patients with IBD are significantly above those in other types of colitis, inactive IBD disease, or normal controls. Furthermore, the growth expression of IL-8 in patients with IBD may perpetuate or initiate IBD by interactions with TNF-α and/or IL-1 in neutrophil activation [37]. IL-8 was induced more in LPS-exposed HT-29 cells than other cells [38]. We examined the inhibitory effect of IL-8, IL-1β, and IL-6 expression on R and found that R reduced IL-8 levels in LPS-induced HT-29 cells (Figure 8) and inhibited IL-1β, IL-6 levels in LPS-induced RAW 264.7 cells (Figure 6). These results show that R, isolated from *N. domestica*, has possibility in the treatment of IBD.

In conclusion, our results show that R downregulates NO production in LPS-exposed RAW 264.7 cells. The inhibitory activity of R occurred by the suppression of iNOS and COX-2 expression via the reduction of NF-κB activation. This anti-inflammatory effect was linked to MAPK signaling. R has an inhibitory effect on the activation of phosphate-ERK1/2. In addition, we confirmed that R suppresses pro-inflammatory cytokines, IL-1β and IL-8. Based on the these results, for R to be a latent therapeutic agent of IBD, the therapeutic effects of R isolated from *N. domestica* against IBD will need to be studied using animal models.

## 4. Materials and Methods

### 4.1. Plant Material

The *N. domestica* fruits were collected from the Nambu forest inside Seoul National University, Beagwoon Mountain, Gwangyang city, Jeollanam-do, Korea on January 2017. A voucher specimen (SCNUP 20) was deposited to the laboratory of pharmacognosy, College of Pharmacy, Sunchon National University, Suncheon-si, Jeollanam-do, Korea.

### 4.2. Extraction and Isolation

The dried fruits of *N. domestica* (347.1 g) were pulverized and their methanol extracts were obtained by sonication at room temperature (1.5 h × 4). The fruit extracts were suspended in water and partitioned successively with *n*-hexane, ethyl acetate (EtOAc), *n*-butanol, and water, resulting in solid residues weighing 0.6 g, 1.6 g, 7.4 g, and 11.0 g, respectively. The EtOAc fraction of *N. domestica* fruits was separated using an MPLC (YMC-DispoPack AT SIL-25, YMC, Kyoto, Japan, silica gel 40 g; CHCl_3_:CH_3_OH = 95:5 → CH_3_OH; 15 mL/min) to obtain five fractions (E1 to E5). Among them, E3 was subjected to HPLC (YMC J’sphere ODS-H80, YMC, C_18_, 250 × 10 mm; H_2_O:CH_3_CN = 95:5 → CH_3_CN; 2 mL/min) to yield ten fractions (E3-1 to E3-10). Robustaflavone (1.2 mg, *t*_R_ 11.2 min) was derived from the E3-1 fraction. 

*Robustaflavone*: Yellow powder; C_30_H_18_O_10_; ^1^H NMR (400 MHz, CD_3_OD): δ 8.08 (1H, d, *J* = 1.6 Hz, H-2′), 7.93 (1H, dd, *J* = 8.4, 2.0 Hz, H-6′), 7.60 (2H, d, *J* = 8.8 Hz, H-2‴,6′′′), 7.12 (1H, d, *J* = 7.2 Hz, H-5′), 6.67 (2H, d, *J* = 8.4 Hz, H-3′′′,5′′′), 6.64 (1H, s, H-3), 6.62 (1H, s, H-3′′), 6.33 (1H, s, H-8′′), 6.30 (1H, d, *J* = 2.0 Hz, H-8), 6.17 (1H, d, *J* = 1.2 Hz, H-6); ^13^C NMR (100 MHz, CD_3_OD): δ 182.0 (C-4′′), 181.3 (C-4), 166.4 (C-7), 165.8 (C-2′′), 162.5 (C-7′′), 162.1 (C-2), 161.1 (C-5), 160.9 (C-4′′′), 160.3 (C-5′′), 159.8 (C-4′), 159.4 (C-9), 157.3 (C-9′′), 132.8 (C-2′), 129.3 (C-2′′′,6′′′), 127.4 (C-6′), 122.2 (C-1′′′), 121.3 (C-1′), 120.6 (C-3′), 117.9 (C-5′), 116.8 (C-3′′′,5′′′), 104.8 (C-6′′), 103.7 (C-10,10′′), 103.2 (C-3,3′′), 100.2 (C-6), 95.1 (C-8), 94.4 (C-8′′). The spectra are available as Supplementary Materials.

### 4.3. Cell Culture

The mouse macrophage cells (RAW 264.7) and human colonic epithelial cells (HT-29) were purchased from the Korean cell line bank (Seoul, Korea). These cells were grown in Dulbecco’s modified Eagle’s medium (DMEM) supplemented with 10% heat-inactivated fetal bovine serum (FBS) and a 100 IU/mL penicillin and 100 µg/mL streptomycin solution (HyClone, Logan, UT, USA) at 37 °C in a 4.5% CO_2_-containing humidified atmosphere.

### 4.4. Cell Viability Assay

RAW 264.7 macrophage cells were plated at a density of 1 × 10^5^ cells/well in 96-well plates. After 24 h, these cells were treated with various concentrations of the samples for 24 h. The cytotoxic effect of the samples on the cells was evaluated using the MTT (Sigma-Aldrich, Saint Louis, MO, USA) assay. The cultured cells were incubated with MTT at 37 °C (0.05 mg/mL). After 4 h, the supernatant was removed by suction and 100 µL DMSO was added to dissolve the formazan crystals. Absorbance was measured at 570 nm using a microplate reader. 

### 4.5. Measurement of NO Production

RAW 264.7 macrophage cells were incubated for 1 h with the prepared samples and then treated with 1 µg/mL LPS for 24 h. The cultured cells (1 × 10^5^ cells/well) were added to an equal amount of Griess reagent (equal volumes of 1% (*w*/*v*) sulfanilamide in 5% (*v*/*v*) phosphoric acid and 0.1% (*w*/*v*) naphtylethylene) and incubated at RT for 10 min; the absorbance was measured at 550 nm using a microplate reader (BioTek Instruments, Inc., Winooski, VT, USA). Serum-free culture medium was used as control and the nitrite production was measured.

### 4.6. Western Blot Analysis

RAW 264.7 macrophage cells were washed twice with ice-cold phosphate-buffered saline (PBS). The washed cells were harvested and the proteins were extracted using lysis buffer (50 mM Tris, pH 7.4, 2 mM EDTA, 0.1% Triton X-100, 1 mM PMSF, 25 µg/mL leupeptin, and 20 µg/mL pepstatin). The protein concentration was measured using the Bio-Rad Protein Assay Reagent (Bio-Rad, Hercules, CA, USA). Samples, containing 30 μg of protein each, were electrophoresed on 10% SDS-PAGE gels and blotted onto PVDF membranes. The membranes were blocked for 1 h in 5% skim milk in plain buffer (20 mM Tris (pH 7.4), and 136 mM NaCl). The membranes were incubated with primary antibodies—to detect COX-2, iNOS, NF-κB, and pERK1/2 proteins—overnight at 4 °C, following which the membranes were incubated in horseradish peroxidase (HRP)-conjugated secondary antibodies for 2 h at room temperature with shaking. The protein bands were detected with enhanced chemiluminescence detection reagents (Thermo Fisher Scientific, Waltham, MA, USA) and visualized using a Bio imaging-system (MicroChemi 4.2 Chemilumineszenz-System, Neve Yamin, Israel).

### 4.7. Measurement of Cytokine Production

The levels of IL-1 and IL-6 in the RAW 264.7 macrophages and IL-8 in the HT-29 cell cultures were quantified by ELISA kits (BD OptEIA^TM^, San Diego, CA, USA) following the manufacturer’s instructions.

### 4.8. Statistical Analysis

The experimental data are expressed as the mean ± standard deviation (SD). The data used were tested through at least three independent experiments. Statistical analysis was done using one-way ANOVA to compare multiple group means followed by the Student’s *t*-test; significance was considered at *p* < 0.05.

## Supplementary Materials

The following are available online, S1. ^1^H and ^13^C NMR spectra of R; S2. ^1^H–^1^H COSY spectrum of R; S3. HMBC spectrum of R.
