# Supplementary material for: Robustaflavone Isolated from Nandina domestica Using Bioactivity-Guided Fractionation Downregulates Inflammatory Mediators
Source: Molecules. 2019 May 8;24(9):1789. doi: 10.3390/molecules24091789 (PMC6540067; doi:10.3390/molecules24091789)

Supplementary data

Robustaflavone Isolated from *Nandina domestica* Using Bioactivity-Guided Fractionation Downregulates Inflammatory Mediators

Ara Jo, Hyun Ji Yoo and Mina Lee*

College of Pharmacy, Sunchon National University, 255 Jungangno, Suncheon-si 57922, Jeonnam, Republic of Korea

Supporting Information Available

S1. ^{1}H and ^{13}C NMR spectra of R

S2. ^{1}H- ^{1}H COSY spectrum of R

S3. HMBC spectrum of R

S1. ^{1}H NMR spectra of R

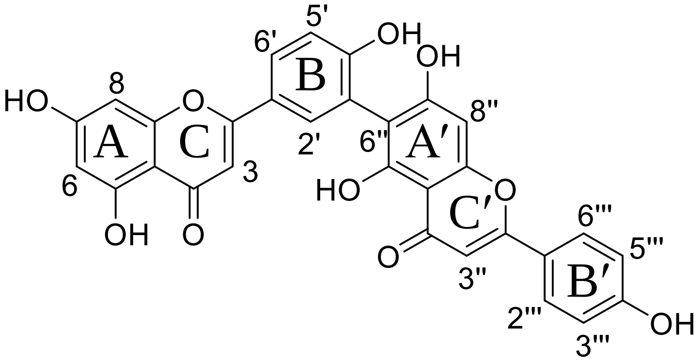

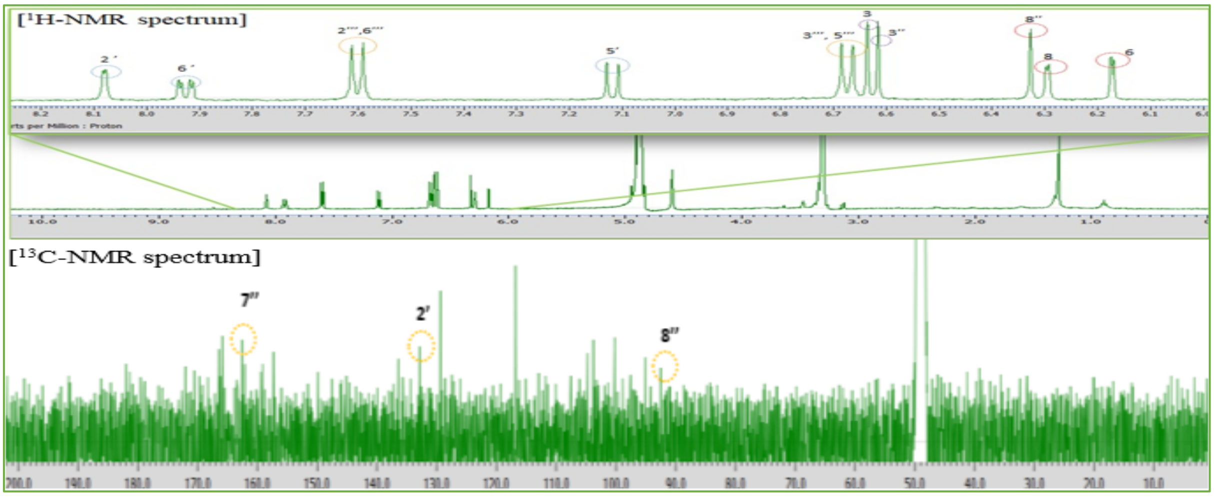

S2. ^{1}H-^{1}H COSY and HMBC spectrum of R

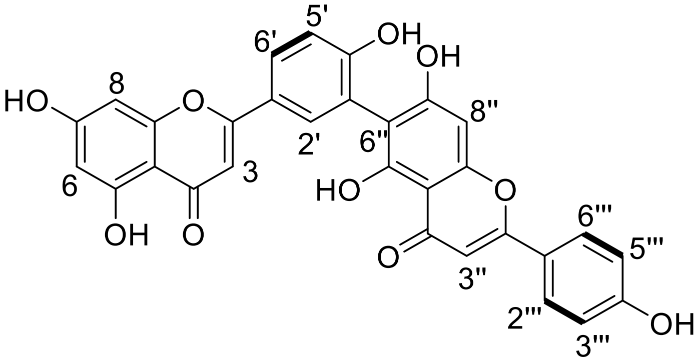

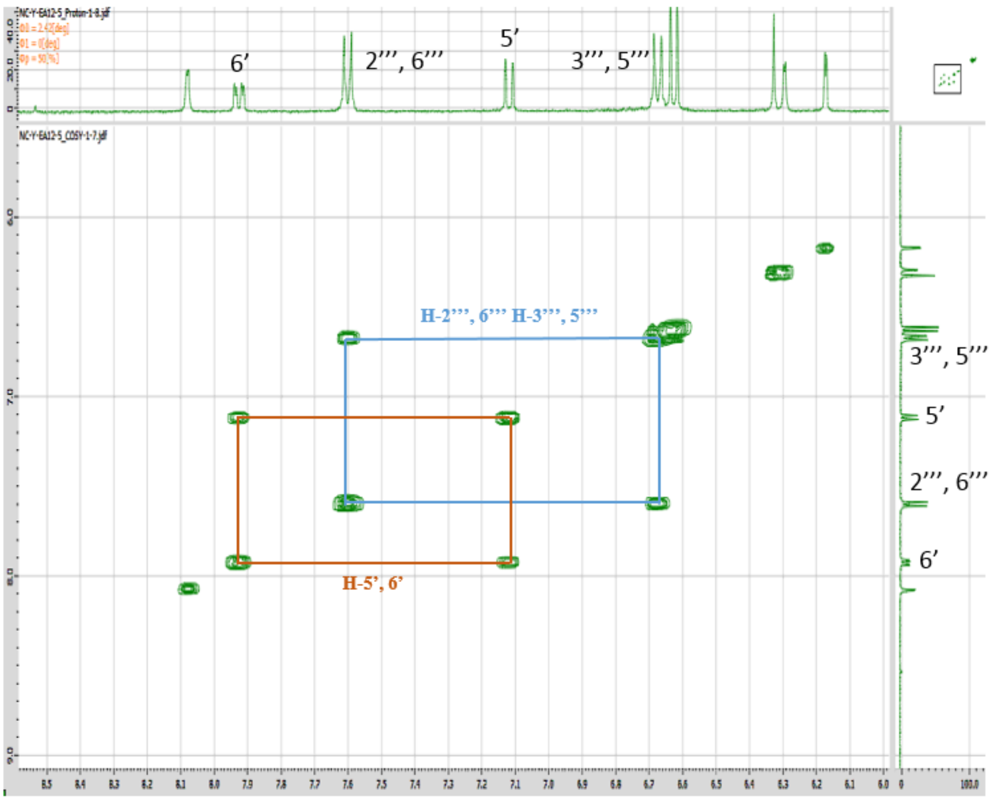

S3. HMBC spectrum of R

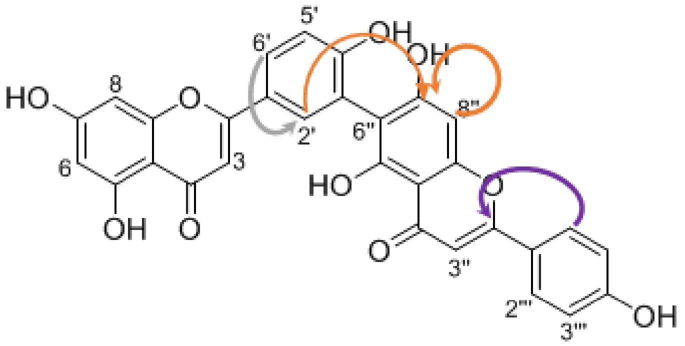

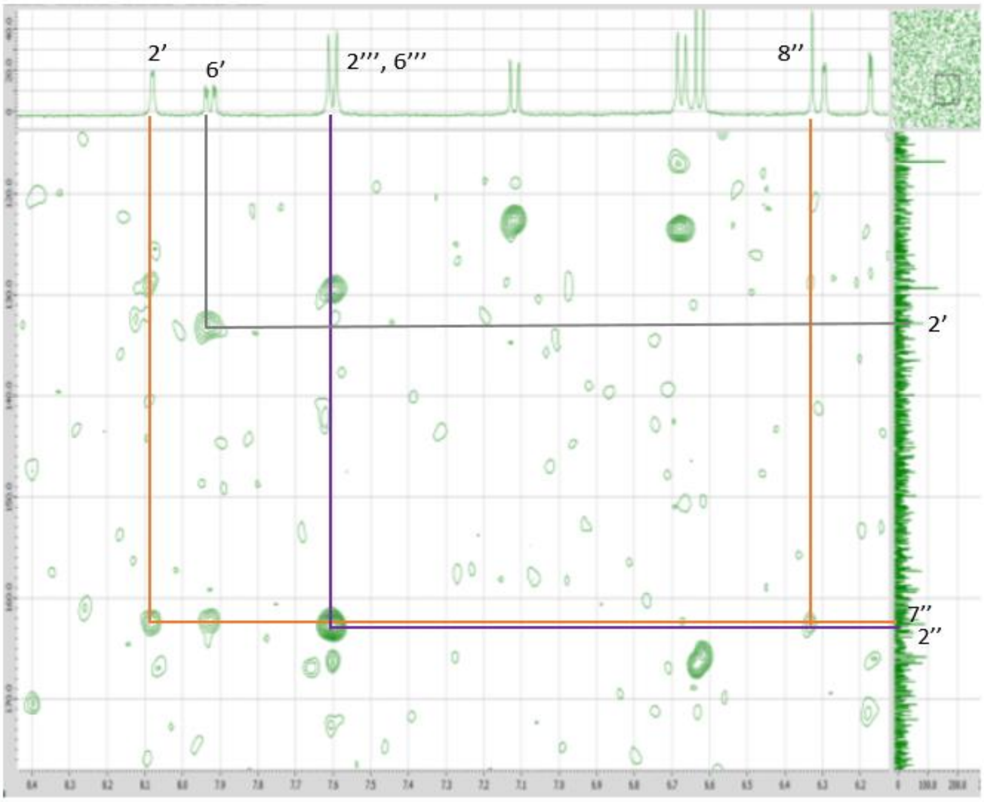

Supplement: Supplementary file 1 [file molecules-24-01789-s001.pdf]
